# Auxotrophy to Xeno-DNA: an exploration of combinatorial mechanisms for a high-fidelity biosafety system for synthetic biology applications

**DOI:** 10.1186/s13036-018-0105-8

**Published:** 2018-08-14

**Authors:** Christopher M. Whitford, Saskia Dymek, Denise Kerkhoff, Camilla März, Olga Schmidt, Maximilian Edich, Julian Droste, Boas Pucker, Christian Rückert, Jörn Kalinowski

**Affiliations:** 1 Center for Biotechnology, Bielefeld University, 33615 Bielefeld, Germany; 2 Faculty of Biology, Bielefeld University, Bielefeld, Germany; 3 Present address: Evolution and Diversity, Department of Plant Sciences, University of Cambridge, Cambridge, UK

**Keywords:** Kill switch, iGEM, Semantic containment, Physical containment, Auxotrophy, *Escherichia coli*, BioBrick, Genetic engineering

## Abstract

**Background:**

Biosafety is a key aspect in the international Genetically Engineered Machine (iGEM) competition, which offers student teams an amazing opportunity to pursue their own research projects in the field of Synthetic Biology. iGEM projects often involve the creation of genetically engineered bacterial strains. To minimize the risks associated with bacterial release, a variety of biosafety systems were constructed, either to prevent survival of bacteria outside the lab or to hinder horizontal or vertical gene transfer.

**Main body:**

Physical containment methods such as bioreactors or microencapsulation are considered the first safety level. Additionally, various systems involving auxotrophies for both natural and synthetic compounds have been utilized by iGEM teams in recent years. Combinatorial systems comprising multiple auxotrophies have been shown to reduced escape frequencies below the detection limit. Furthermore, a number of natural toxin-antitoxin systems can be deployed to kill cells under certain conditions. Additionally, parts of naturally occurring toxin-antitoxin systems can be used for the construction of ‘kill switches’ controlled by synthetic regulatory modules, allowing control of cell survival. Kill switches prevent cell survival but do not completely degrade nucleic acids. To avoid horizontal gene transfer, multiple mechanisms to cleave nucleic acids can be employed, resulting in ‘self-destruction’ of cells. Changes in light or temperature conditions are powerful regulators of gene expression and could serve as triggers for kill switches or self-destruction systems. Xenobiology-based containment uses applications of Xeno-DNA, recoded codons and non-canonical amino acids to nullify the genetic information of constructed cells for wild type organisms. A ‘minimal genome’ approach brings the opportunity to reduce the genome of a cell to only genes necessary for survival under lab conditions. Such cells are unlikely to survive in the natural environment and are thus considered safe hosts. If suitable for the desired application, a shift to cell-free systems based on Xeno-DNA may represent the ultimate biosafety system.

**Conclusion:**

Here we describe different containment approaches in synthetic biology, ranging from auxotrophies to minimal genomes, which can be combined to significantly improve reliability. Since the iGEM competition greatly increases the number of people involved in synthetic biology, we will focus especially on biosafety systems developed and applied in the context of the iGEM competition.

## Background

Safety approaches in synthetic biology are frequently distinguished into mechanisms of biosecurity or biosafety. However, many methods can only be distinguished at a theoretical level into biosafety or biosecurity [1]. Here, we focus on biosafety, defined as the combination of all preventive measures against accidental infection with, or release of, genetically engineered organisms into the environment [2]. Nevertheless, some of the presented mechanisms can also contribute to biosecurity, defined as the protection of biological systems against an intended misuse [3].

Recombinant DNA technology has been applied since 1973 to modify the genetic information of cells for scientific as well as for economic purposes [4]. However, this technology poses risks if applied carelessly [5] or with deleterious intent [6–8]. This risk has long been recognized, and multiple safety regulations have been proposed to prevent harm to humans, animals, and the environment [9, 10].

As *Escherichia coli* became a model organism in molecular biology and biotechnology, the development of safety strains was of high interest [11]. For example, *E. coli* K12 MG1655 [12, 13] and *E. coli* B derivatives like REL606 and BL21 (DE3) are safety strains [14] commonly used for molecular cloning and heterologous gene expression [15–19]. Due to several mutations, these strains are no longer able to compete with wild type strains within the human gut [5, 20]. More sophisticated strains have been developed, including dedicated biosafety approaches like the *relA* deletion in combination with a conditional *phoA* expression [21, 22], as described in the next chapter. The combination of different precautions may have successfully prevented any accidents involving genetically modified organisms (GMOs) during the last four decades [2, 23–25]. Today, the handling of GMOs is strictly controlled for multiple reasons, including ecological and health considerations [2, 26, 27], but also the protection of intellectual property [28].

However, the demand for novel biosafety systems is still high, due to the spread of genetic engineering capabilities which in turn is facilitated by the growing number of people involved in the field of synthetic biology. Students participating in the iGEM [29] competition have contributed significantly to the synthetic biology toolbox [30–43]. The increased availability of genome sequences [12, 44] as well as an ever-increasing number of sequenced bacterial genomes could enhance the discovery and implementation of innovative safety systems. We will describe general biosafety mechanisms along with advanced systems developed within this competition. It is our intention to provide an overview of the applied concepts as well as the completely implemented and characterized systems. Since the deletion of an essential gene is probably the most basic mechanism, we will start with the description of auxotrophies. Released cells with failing auxotrophy systems could be stopped by the activation of kill switches. Activation of such mechanism should kill the cell, but the nucleic acids are still present, requiring a self-destruction of the genetic information to prevent horizontal gene transfer (HGT). Given that all known life on earth is based on the same biochemistry, cells are composed of common materials like proteins, sugars, lipids, and nucleic acids. Although the sequence of nucleotides and amino acids varies within higher layer molecules, the basal molecules are shared across all species. A common genetic code allows the exchange of genetic information between different species through HGT. In general, HGT might not be beneficial for a specific species [45] but is assumed to maintain an overall unity of life by preventing changes in the genetic code [46]. Therefore, central signal and metabolic pathways are shared between distantly related species, while additional pathways are specific to certain taxonomic groups. Interesting in terms of biosafety are rare cases, where replicating systems escaped this system and modified their genetic code [47–49]. Modifications to this universal genetic code pose a powerful biosafety mechanism [50]. Therefore, xenobiology-based containment is another way to prevent the successful transfer of information from another species. Sensors for light, temperature, pH, UV or other physical parameters can be used as triggers for biosafety systems. Avoiding issues of vertical gene transfer (VGT) through cell division, cell free systems seem to be a well suited biosafety approach. In combination with xenobiology, cell free systems could efficiently minimize the risk of HGT and VGT. Finally, we aim to describe the design of an optimized biosafety system by integration of different mechanisms, which consequently could be referred to as a synthetic biosafety system [51].

## Auxotrophy

One of the earliest methods applied for biocontainment was auxotrophy, introduced either by random mutation or targeted engineering [52]. In respect to biosafety, auxotrophic organisms are not able to synthesize one or more compounds required for survival or replication, so the missing components must be provided externally [53]. Therefore, the organism cannot survive outside a controlled environment, such as a bioreactor. Research on auxotrophic strains dates back to 1941, when Beadly and Tatum characterized a strain of *Neurospora sitophila* without pyridoxine [54]. The first construction and application of an auxotroph *E. coli* strain for biosafety reasons was developed in 1977 by Curtiss and colleagues [52], who developed the strain χ1776, which requires diaminopimelic acid (DAP) and thymine or thymidine for growth. Indeed, many prominent laboratory *E. coli* strains are auxotrophic mutants [55]. Over the years, auxotropy-based systems have been developed further and successfully improved with regards to reducing the escape frequency [53] (the escape frequency describes the probability of an organism bypassing the containment measures and is usually determined by quantification of escape mutants found in a defined cell number [56]), with an increasing number of studies reporting escape frequencies (Table 2) below the detection limit (e.g. [57, 58]). Auxotrophy-based systems are manifold in nature, with different hosts (e.g. *E. coli* or other bacteria, plants, or fungi) and various possible dependencies, most commonly for amino acids or vitamins.

### Strains auxotrophic for proteinogenic amino acids

The dependency of engineered organisms on one or multiple naturally occurring amino acids has been used in many studies to improve biosafety via containment or replacement of problematic functions. A prime example for the latter is the substitution of antibiotic resistance markers on plasmids. These standard tools in genetic engineering traditionally contain an antibiotic resistance gene as a selection marker, which further ensures plasmid maintenance. This is potentially hazardous, as the resistance genes could be released into the environment [59–61]. As an alternative to antibiotic selection markers, the auxotrophic *E. coli* strain M15 carries an inactive *glyA* gene, which is necessary for intracellular glycine synthesis [59]. The antibiotic-free expression system utilizes the M15 strain with a constructed plasmid providing the *glyA* gene and thereby preventing plasmid loss. A similar approach was used in the modified *E. coli* strain JM83 auxotrophic for proline and a complementing vector supplying the *proBA* genes necessary for the proline biosynthetic pathway [62]. Antibiotic resistance-free systems have also been used as a safe method for vaccine production and are not limited to *E. coli*. An example is the cattle vaccine *Brucella abortus* mutant strain (RB51) with non-functional 3-isopropylmalate dehydrogenase (*leuB*), which is essential for biosynthesis of leucine [60]. By adding a plasmid carrying the wild type *leuB* to the leucine-auxotrophic strain, the *B. abortus* strain is able to survive and the corresponding plasmid is maintained due to selective pressure. The resulting system is a biologically safe vaccine system, as the *B. abortus* mutant strain induces immune responses through a plasmid containing the gene encoding the desired antigen, but no antibiotic resistance gene, which could potentially spread to the microbial flora of an animal. In 2012, a system was engineered that supported the usage of the duckweed *Lemna* for the production of vaccines and therapeutics [63]. An avian influenza vaccine antigen was successfully expressed in the system containing an isoleucine auxotroph strain of *Lemna*. Particularly, threonine deaminase expression necessary for the isoleucine biosynthesis had been inactivated in a system containing an isoleucine auxotroph strain of *Lemna*, which was then supplemented with isoleucine to enable growth.

### Strains auxotrophic for other natural components

A different biosafety approach is to prevent an organism from building a cell wall by means of engineered auxotrophy. For example, D-alanine is an essential component of the peptidoglycan layer, since it is one of the molecules necessary for the cross-linkage of the polysaccharide chains [64, 65]. L-alanine can be converted to D-alanine by two different alanine racemases in *E. coli*, one being encoded by *alr*, the other one by *dadX* [66]. In Corynebacteriaceae, only the *alr* gene is present, which was deleted in *Corynebacterium glutamicum* [65] and *Mycobacterium smegmatis* [67, 68]. While Tauch et al. [65] successfully created a D-alanine auxotroph and subsequently used a plasmid-borne *alr* gene to replace an antibiotic resistance marker, Chacon et al. [67] found that *M. smegmatis* mutants with inserted inactivated D-alanine racemase gene were still able to grow without supplied D-alanine.

Focusing on the aspartate semialdehyde dehydrogenase (*asd*) gene, an antibiotic resistance gene free plasmid *Salmonella enteritidis* “ghost” (i.e. an empty cell envelope possessing intact bacterial surface structures and integrated antigen proteins) has been developed as a safe vaccine against infectious diseases in chicken [69]. The strategy was to delete the *asd* gene, which encodes aspartate ß-semialdehyde dehydrogenase (Asd), an enzyme located at the root of biosynthesis of the aspartate-derived amino acids lysine, threonine and methionine. Asd is also involved in forming the precursor for the production of diaminopimelic acid (DAP), which in turn is necessary to build the cell wall structures in most bacteria. As a complement, a plasmid carrying the *asd* gene and a lysis system (pJHL101) for production of the “ghost” was constructed. The resulting strain was tested as a vaccine in chickens and shown to elicit substantial immune responses [69].

Additionally, an auxotrophic *E. coli* strain has been engineered by inactivating the chromosomal quinolinic acid phosphoribosyltransferase (QAPRTase) gene, a key enzyme in the nicotinamide adenine dinucleotide (NAD) synthesis pathway [61]. Thus, an antibiotic-free plasmid selection system has been created by complementing the system with a plasmid containing the QAPRTase gene of the mouse. The resulting antibiotic-free selection system was the first to use a vertebrate gene and the strain can be maintained in complex (LB) or minimal media.

Hirota et al. developed a novel biocontainment strategy based on a dependency on phosphite [70]. By disrupting all endogenous P_i_ and organic P_i_ transporters and by producing the transporter HtxBCDE, as well as HtxA and PtxD, the researchers engineered an *E. coli* strain dependent on phosphite or hypophosphite. HtxBCDE, a transporter from *Pseudomonas stutzeri* WM88, is capable of transporting phosphite and hypophosphite but not phosphate. Production of the hypophosphite dioxygenase HtxA and of the phosphite dehydrogenase PtxD allowed utilization of hypophosphite and phosphite as sole sources of P_i_. When tested on non-permissive growth medium, no escape mutants were detected after 21 days. An escape frequency of 1.94 × 10^− 13^ was achieved, which is extremely low compared to other biocontainment approaches (Table 2).

Examples of natural containment systems also include a strategy to treat inflammatory bowel disease in humans by a genetically modified *Lactococcus lactis* strain. The strain was altered by replacing *thyA*, the gene encoding thymidylate synthase, which is essential for growth of the bacterium, with a human interleukin-10 gene [71]. The resulting strain produces human interleukin-10, which is used as a therapeutic for inflammatory bowel disease. Furthermore, it is dependent on thymidine or thymine for survival, thus providing a biologically contained system for clinical treatment in humans. The strain has been successfully tested in a clinical trial [72]. Specifically, no severe side effects were detected in the patients, and the *thyA*-deficient organism was found to be unable to replicate without proper supplementation.

To increase performance, auxotrophic systems can be combined to engineer strains with multiple auxotrophies in order to further improve containment capability, such as the synthetic *E. coli* auxotrophs with ligand-dependent essential genes (SLiDE) [73], which uses a mutation approach demonstrated by the Karanicolas lab [74]. The resulting strains can only survive if they are supplied with the low-cost ligand benzothiazole, enabling the growth through the gain-of function of an essential protein in which an aromatic amino acid residue was mutated to a glycine. For further improved containment, multiple SLiDE alleles were incorporated in *E. coli*. While escape frequencies (Table 2) for strains with a single SLiDE allele ranged between 8 × 10^− 4^ and 3 × 10^− 9^, incorporating two alleles resulted in improved escape frequencies of up to 5 × 10^− 10^, and building in three SLiDE alleles lead to undetectable escape frequencies below 3 × 10^− 11^ [74].

Still, even combining several auxotrophies for natural compounds required by the organisms cannot overcome a major drawback inherent in this approach: The necessary compounds can potentially occur in the natural environment, compromising the safety mechanism. Hence, compounds which do not naturally occur in the environment have been considered as a complement for auxotrophs.

### Evaluation of auxotrophic strains for biosafety purposes

**Fig. 1:**
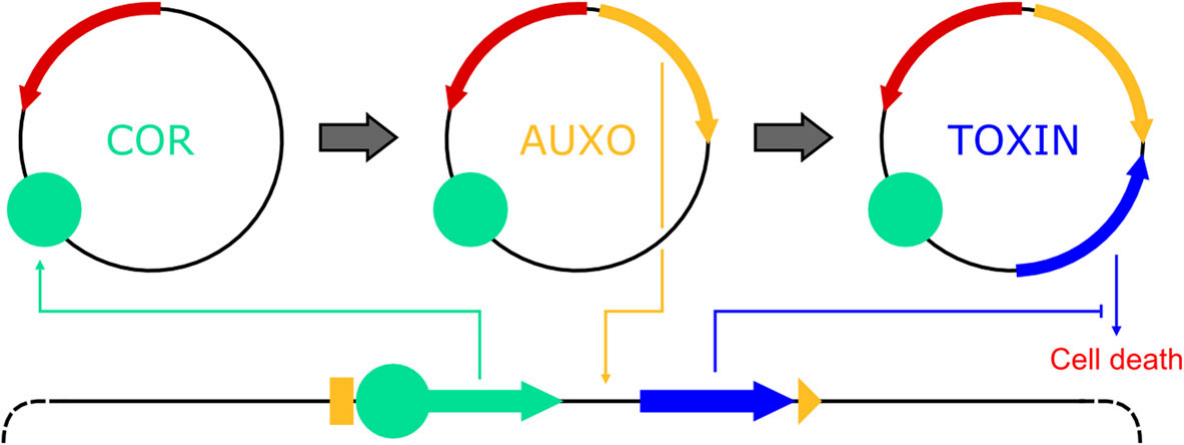
*GeneGuard* as an example for a modular, multilayered biosafety system [32]. The plasmid system uses conditional origins of replication, auxotrophies and TA systems. Replication initiators are provided *in trans* by the host, minimizing the risk of replication in unintended hosts. The host lacks the essential genes *thyA* or *dapA* which are located on the plasmid, making the plasmid essential for cell survival. As a third layer the TA systems Epsilon-Zeta or Kis-Kid were used to kill the cells after losing the plasmid

In conclusion, auxotrophic GMOs are simple and cost-efficient biocontainment systems with a variety of possible applications. Complementing substances can be provided via the growth media, and multiple auxotrophies further reduce escape risks. However, auxotrophic systems suffer from substantial drawbacks, such as possible metabolic cross-feeding, toxic overexpression, and loss of function due to decreased selection pressure in a heterogeneous natural environment (for a summary see [75]). Therefore, instead of relying on an auxotrophy system alone, they are often part of a complex multilayered biosafety system, such as *SafeGuard* [76] or *GeneGuard* [32]. In the *GeneGuard* plasmid system (Fig. 1), auxotrophies based on translocation of the essential genes *thyA* or *dapA* to a plasmid location were combined with a conditional origin of replication and toxin-antitoxin system to engineer a host-plasmid mutual dependency [32].

### Auxotrophy systems in the iGEM competition

Compared to their prominence in research, the implementations of auxotrophic strains in iGEM projects have been relatively rare, which is in part due to the fact that the iGEM foundation does not collect and provide such genetically modified strains, making it necessary for the iGEM teams to either create them themselves or obtain them from other sources.

A successful implementation of an auxotrophic strain for a biosafety system had been accomplished by team Bielefeld-Germany 2013 [77]. The team created a biosafety system composed of three independent layers, one of which being a D-alanine auxotrophy, established by deleting both racemase genes, *alr* and *dadX*. The team showed that cells grown on media without D-alanine supplemented or not carrying a plasmid-bound alanine racemase (BBa_K1172901) were unable to grow. Bielefeld-CeBiTec 2014 [78] adapted the system for an antibiotic-free selection, hence eliminating the problems resulting from extensive use of antibiotics in laboratories. This underlines the multifaceted possible applications of auxotrophies.

Other teams have conceptually considered auxotrophic strains for their projects, whilst not implementing these themselves. Team BYU Provo 2014 [79], for example, worked with the bacterium *Nitrosospira multiformis* in the context of wastewater treatment and identified serine as being abundant in the bioreactor/sedimentation tank, but not in the waterways. Therefore, it was proposed that deletion of *serA* from *N. multiformis* would result in a dependency for serine, hence making growth outside the bioreactor or sedimentation tank improbable.

The biosafety system for *Synechocystis* sp. suggested by team Amsterdam 2015 [80] is based on an arginine and proline auxotrophy. To implement the auxotrophies, deletion of *argH* for arginine and *proC* for proline auxotrophy was proposed. Since the team could not finish the desired work in time, no results regarding the effectiveness of a combination of an arginine and proline auxotrophy are available.

## Kill switch: plasmid retention and cell destruction

While auxotrophies prevent a continuous survival or replication of cells, biological kill switches are applied to prevent the survival of a cell immediately. Typically, these biosafety systems make use of bacteriolytic toxin-antitoxin systems (TA) of different complexities. So far, five different types of TA systems have been identified and described in the literature [81].

Antisense RNA meditates the inhibition in type I systems [82, 83]. Besides the sense strand transcription of the toxin gene, the sequence of the reverse strand is partially transcribed as well, leading to a complementary RNA called an antitoxin. This antisense transcript binds to the sense transcript and prevents the translation by blocking off the ribosome or by promoting the degradation of the mRNA, respectively [84, 85]. Famous examples of type I TA systems are *hok-sok* and similar systems like *pndA-pndB* and *symR-symE* [86–89].

Type I TA systems have not been commonly applied in the iGEM competition in the context of biosafety (Table 1). Most teams have integrated type I TA systems for plasmid maintenance and as an alternative for antibiotic resistance cassettes. Team NTU Taida 2012 [90] used the Hok-Sok homologue SrnB-SrnC (BBa_K817015) to ensure plasmid maintenance and to kill cells after plasmid loss. University of Maryland 2015 [91] investigated the ability to use Hok-Sok (BBa_K1783001) for antibiotic-free plasmid maintenance.

**Table 1:** Biosafety parts in the iGEM competition

Team	Mechanism	Organism	Characteristics	BioBrick	Status ^a^	References
NTU Taida 2012	Type I TA-system	*E. coli*	SrnB-SrnC TA-system, used for plasmid maintenance	BBa_K817015	Available	[90]
University of Maryland 2015	Type I TA-system	*E. coli*	Hok-Sok TA-system, antibiotic free plasmid maintenance	BBa_K1783001	Not available	[91]
Paris Bettencourt 2012	Type II TA-system	*E. coli*	Col E2 TA-system, located on two separate plasmids	BBa_K914001, Ba_K914002	Available, complicated	[104]
Wageningen UR 2014	Type II TA-system	*E. coli*	Combination of the Kis-Kid and the Epsilon-Zeta TA-system to prevent horizontal gene transfer, located on two separate plasmids	BBa_K1493601, BBa_K1493603	Available, complicated	[105]
UC Berkeley 2008	Type II TA-system	*E. coli*	Holin-antiholin TA-system, coupled expression of lysozyme	BBa_K112808	Complicated	[106]
LMU Munich 2012	Sporulation induced killswitch	*B. subtilis*	*mazF* under control of P_ydfG_, which is activated in germinating cells	BBa_K823044	Available	[113]
TU Eindhoven 2014	Timer-coupled killswitch	*E. coli*	Oscillating concentration of Spo0A^P^ induces expression of a toxin		Conceptional	[114]
UC Berkley 2007	RNase	*E. coli*	CDS of Barnase, without start codon	BBa_I716211	Available	[124]
Bielefeld Germany 2013	RNase	*E. coli*	CDS of Barnase	BBa_K1172904	Available	[77]
UC London 2012	Sequence specific self-destruction system	*E. coli*	threefold active biological containment system containing *EcoRI*/*Eco*RI methyltransferase, Holin/Anti-Holin Endolysin and Colicin-E3/Colicin Immunity E3	BBa_K729009, BBa_K729010	Not available	[133]
TU Munich 2013	Sequence unspecific self-destruction system	*Physcomitrella patens*	Mature Nuclease NucA from *Staphylococcus aureus* (Thermonuclease) in RFC [25]	BBa_K1159105	Available	[138]
HKU Hongkong 2015	Sequence specific and unspecific self-destruction system	*E. coli*	CRISPR/Cas9 containment device repressed by arabinose and tryptophan	BBa_K1774000	Not available	[160]
Harvard 2010	Genetic fence	*E. coli*	Barnase: gene for the genetic fence, Barstar: Inhibitor of barnase	−/−	Conceptional	[374]
Virginia 2016	Dependency on a modified amino acid	*E. coli*	N-carbobenzyloxy (CBZ)-cleavage enzyme to detach the protecting group frim amino acids	BBa_K1879000	Not available	[375]
Virginia 2016	Dependency on a modified amino acid	*E. coli*	Mutatnt Leucyl-tRNA synthetase	−/−	Conceptional	[375]
Bielefeld-CeBiTec 2015	Cell free protein synthesis	*E. coli* extract	Mixture of amino acids, co factors, cell extract, NTPs, energy source, DNA template, Mg- and K-glutamate solutions, nuclease free water for cell free protein synthesis in microcentrifuge tubes or a multi-well plate	−/−	−/−	[280]
Freiburg 2015	DiaMIX	*E. coli* extract	Cell-free expression mix for reaction in a microfluidic chamber	−/−	−/−	[284]
Paris Saclay 2015	Microencapsulation		Silica beads			[302]
Paris Bettencourt 2012	Microencapsulation		Alginate beads			[104]
Paris Saclay 2015	RNA-Thermometer	*E. coli*	Thermosensitive cI repressor (cI857) is placed under the control of the RNA thermometer ROSE	BBa_K1707013	Not available	[302]
TU Delft 2008	RNA-Thermometer	*BradirhizobiumJaponicum*, *E. coli*	ROSE-RNA Thermometer	BBa_K115001	Available	[346]
TU Delft 2008	RNA-Thermometer		ForU RNA-Thermometer	BBa_K115002	Available	[346]
NCTU Taiwan 2011	RNA-Thermometer	*E. coli*	Promoter (LacI regulated) + RNA thermometer+*vioD* with RNA thermometer+tetR+double terminator	BBa_K539461	Available	[347]
NCTU Taiwan 2011	RNA-Thermometer	*E. coli*	Promoter (LacI regulated) + alss+*ilvC*+*ilvD* (each preceded by own RBS) and RNA thermometer+terminator	BBa_K539691	Available	[347]
NCTU Taiwan 2011	RNA-Thermometer	*E. coli*	a RBS (B0030) +* ilvC *with a RBS (B0030) + *ilvD*	BBa_K539642	Available	[347]
NCTU Taiwan 2011	RNA-Thermometer	*E. coli*	a RBS (B0030) + *ilvD* with RNA thermometer+tetR+terminator	BBa_K539653	Available	[347]
NCTU Taiwan 2011	RNA-Thermometer	*E. coli*	a RBS (B0030) + *ilvC*+a RBS (B0030) +* ilvD* with RNA thermometer+tetR+terminator	BBa_K539674	Available	[347]
METU Ankara 2011	RNA-Thermometer	*E. coli*	Cell destruction via lysis casette		Conceptional	[376]
Cornell 2011	Light-sensor (green)	*E. coli*	CCaS and CCar are integral proteins involved in the green light-induced gene expression	BBa_K597105	Not available	[368]
NYMU Taipei 2014	Light sensor (blue)	*E. coli*	FixK2 blue light sensitive promoter for *ccdB* expression		Conceptional	[377]
Minnesota 2014	Light sensor (blue)		Blue light induced promotor with kill-switch gene be Endolysin and Holin		Conceptional	[378]
HNU China 2014	Light sensor (blue)		Blue light induced casp3 expression		Conceptional	[379]
Braunschweig 2014	Light sensor blue		Blue light leads to the dimerization of the VVD Domains inhibiting the transcription initiation		Conceptional	[380]

This table summarizes biosafety systems designed by iGEM teams. Not all systems make use of BioBricks, some are based on auxotrophic strains or cell-free formulation of a system. Since only BioBricks can be submitted to the iGEM Registry of Standard Biological Parts, no entry could be cited for such systems. The status of all BioBricks was adopted from the database. Ambiguous sequencing results might lead to the classification of functional parts as ‘complicated’. Teams are listed in order of mentions in text

^a^Status as stated in the iGEM Registry of Standard Biological Parts. Ambiguous sequencing results might lead to the classification of functional parts as ‘complicated’

Type II systems are based on two genes that are usually adjacent, are oriented head to tail, and form an operon. Systems characterized as type II TA systems include *ccdA-ccdB*, *kis-kid*, *parDE*, *phd-doc*, *mazE-mazF*, and *axe-txe* [92–97]. The encoded toxin and antitoxin form a stable complex, inhibiting the toxin and thereby preventing the deleterious effects on the host cell. Furthermore, the toxin-antitoxin complex binds to the antitoxin-toxin promoter region and represses the transcription of the antitoxin and toxin genes [98–101]. Since the antitoxin is less stable than the toxin, a ‘point of no return’ can be reached under stress conditions, resulting in growth inhibition and, eventually, cell death [96, 97, 102, 103].

Several teams in the iGEM competition have tried to apply the principle of natural type II TA systems to synthetic biosafety systems (Table 1). Team Paris Bettencourt 2012 [104] used a Col E2 toxin and Col E2 antitoxin, encoded on two different plasmids (BBa_K914001, BBa_K914002). Two separate plasmids were used to allow directed degradation of the antitoxin plasmid through a dedicated restriction enzyme system, hence switching on the kill switch.

Team Wageningen UR 2014 [105] used a similar system based on two plasmids. The team applied a combination of the Kis-Kid (BBa_K1493601) and the Epsilon-Zeta (BBa_K1493603) systems to prohibit HGT. Therefore, while the Kis antitoxin and the Zeta toxin were encoded on one plasmid, the Kid toxin and the Epsilon antitoxin were encoded on the other plasmid, creating an interdependent system. In case one plasmid is transferred to a wildtype cell, the analogous antitoxin for the toxin featured on the plasmid is not present, thus killing the recipient.

Team UC Berkeley 2008 [106] used a type II TA system and combined it with a lysozyme (BBa_K112808). The team used holin and antiholin as the toxin-antitoxin pair under control of two separate promoters. While the antiholin is expressed constitutively, expression of the lysozyme and holin is controlled by an inducible promoter. As soon as holin forms pores in the inner membrane, the lysozyme can reach the periplasm and lyse the cell. This system was not designed by the team for biosafety purposes but could be easily adapted for that role.

Type II TA systems have also been extensively applied outside of the iGEM competition. Stirling and colleagues built two evolutionary stable kill switches to control the environment in which a genetically engineered strain of *E. coli* can survive [107]. Their “essentializer” kill switch is based on a bi-stable cI/Cro memory switch. Cell death is induced by loss of the memory switch. The “cryodeath” kill switch was built around a cold-inducible promotor, allowing growth at 37 °C. At a temperature of 22 °C and below, a survival ratio of less than 10^− 5^ was reached (Table 2). Both kill switches were engineered using the type II TA system CcdB-CcdA [107].

**Table 2:** Escape frequencies of selected biosafety systems

Name of the System	Type of System	Escape Frequency	Reference
SLiDE, single allele	Auxotrophy	8 × 10^− 4^ to 3 × 10^− 9^	[69]
SLiDE, two alleles	Auxotrophy	5 × 10^− 10^	[69]
SLiDE, three alleles	Auxotrophy	< 3 × 10^− 11^	[69]
Thymine/Thymidine auxotrophy	Auxotrophy	Below detection limit	[71, 72]
Artificial Phosphite Dependency	Auxotrophy	1.94 × 10^− 13^	[70]
Single ncAA auxotrophy	Auxotrophy/Xenobiology	No escape mutants in >5 × 10^11^ cells	[381]
Triple ncAA auxotrophy	Auxotrophy/Xenobiology	6.41 × 10^−11^	[57]
CcdB	Kill switch	~ 10^− 3^	[28]
*Cryodeath*	Kill switch	< 1 in 10^5^ after 10 days in vivo	[107]
*Deadman*	Kill switch	Below detection limit	[28]
*Passcode*	Kill switch	Below detection limit	[28]
CRISPR mediated DNA degradation	DNA destruction	Viable cells reduced by a factor of 10^8^	[152]
Thermoinduced DNA degradation	DNA destruction	2 × 10^–5^	[135]
*GeneGuard*	Combinatorial system	Below detection limit	[32]
*SafeGuard*	Combinatorial system	<1.3 × 10^− 12^	[76]

In general, the combination of several systems reduces the probability for random mutagenesis to disarm the biosafety system and for cells to bypass the biosafety system. Therefore, multilayered systems like *Passcode*, *Deadman* or *GeneGuard* act as great examples for complex biosafety systems that achieved very low escape frequencies. Engineering artificial auxotrophies, such as an artificial phosphite dependency, can also act as potent biosafety systems, as shown by Hirota et al.

While type I and type II systems are based on interactions of two components of the same kind to mediate inhibition either via RNA or protein toxin-antitoxin complexes, type III systems are based on the interaction of antitoxin RNA with the toxin protein [108, 109]. To our knowledge, only one example for a type III TA system has been identified so far: ToxIN from *Erwinia carotovora* subsp. *atroseptica* which acts as an abortive infection system [108, 110]. No iGEM team has applied this type III TA system as a biosafety system yet.

Recently, type IV and V TA systems have been identified. In type IV systems like *yeeU-yeeV*, the antitoxin and toxin do not form a complex. Instead, the antitoxin acts as an antagonist. While YeeV inhibits the assembly of FtsZ and MreB filaments, YeeU promotes the reaction, hence counteracting the toxicity of YeeV [111]. Type V TA systems like *ghoS-ghoT* also function without formation of a toxin-antitoxin complex. GhoS possesses a sequence-specific endoribonuclease activity, cleaving the GhoT mRNA, thereby inhibiting formation of the toxin protein [112].

Many teams in the iGEM competition used toxins under control of specific promoters without a corresponding antitoxin to create biosafety systems (Table 1). Team LMU Munich 2012 [113] created a kill switch for *Bacillus subtilis* to kill germinating spores (BBa_K823044): the team placed *mazF* under control of P_ydfG_. This promoter is activated by the sigma factor of RNA polymerase ECF41 which is produced during sporulation, thus the team created a sporulation-induced kill switch. Team TU Eindhoven 2014 [114] proposed a timer-coupled kill switch based on the oscillating concentration of phosphorylated Spo0A protein (Spo0AP). Once a certain concentration of Spo0AP is reached, expression of a toxic gene under the control of a Spo0AP-sensitive promoter will be induced, leading to cell death.

The ribonuclease *ba* of *Bacillus amyloliquefaciens* (barnase) and the corresponding inhibitor barstar are sometimes referred to as a toxin-antitoxin system when combined [95]. Whilst the organization of *barstar* and *barnase* genes differ from the organization of natural *toxin* and *antitoxin* systems, the combination of barnase and barstar exhibits many similarities to natural TA-systems. For biosafety applications, the inhibitor gene *barstar* is usually integrated in the chromosome and constitutively expressed. Barnase is encoded on a plasmid, preferably under control of an inducible or repressible promoter. Furthermore, the fusion of the barnase to secretion signals like that of PhoA allows the secretion into the periplasm [115–121]. Secreted barnase can be toxic to bacteria in the proximity of the producing cells if they do not express the inhibitor barstar, but the exact mechanism remains unknown [120, 121]. In combination with another biosafety systems which represses the expression of barnase through arabinose-inducible promoter like P_BAD_, the expression of barnase can be induced if that biosafety mechanism fails, hence killing the host cell [122, 123].

Many iGEM teams employed a barnase system, but only few used it solely for biosafety purposes. Team UC Berkley 2007 [124] was the first to use the barnase (BBa_I716211) under control of the promoter P_BAD_ to induce self-destruction.

Team Bielefeld-Germany 2013 [77] improved the barnase based biosafety system by using it in combination with a D-alanine auxotrophic strain (∆*alr*) as a two-part system (BBa_K1172904). The first part contains the repressor for the promoter of the second part (P_BAD_) and the alanine racemase, both under the control of a rhamnose-inducible promoter (P_Rha_). The second part contains the P_BAD_ promoter and barnase itself. Should the first promoter remain inactive due to auxotrophy failures, barnase is expressed and will lead to cell death.

Team Valencia UPV 2014 [125] aimed to develop a biosafety module to prevent the spread of genetic material in plant seeds. The concept was to use barnase (BBa_I716211) in combination with the tapetum-specific promoter TA29. Due to time restrictions, this concept could not be tested.

**Fig. 2:**
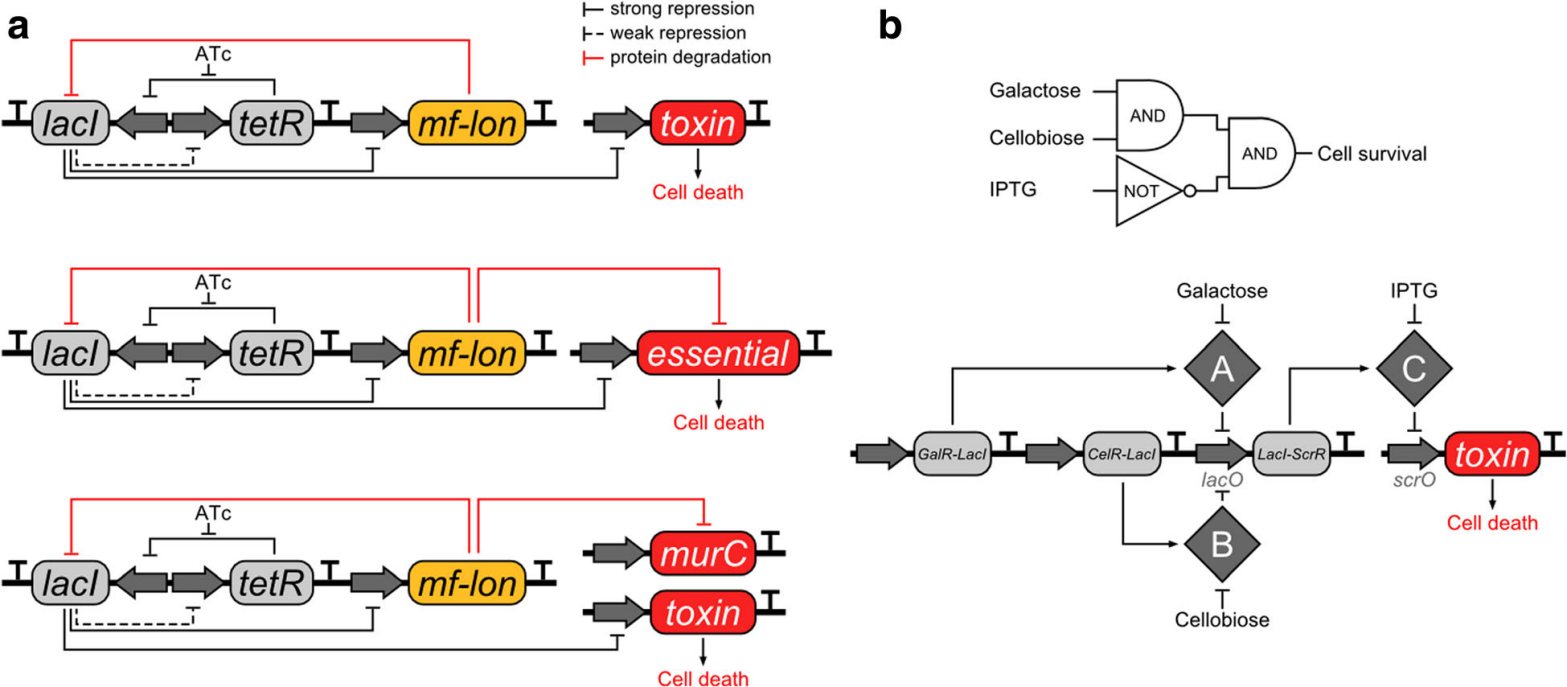
*Passcode* and *Deadman* kill switches [28]. Both systems are built around synthetic molecules which are necessary for the cell’s survival. **a** The *Deadman* kill switch uses anhydrotetracycline (ATc) as the synthetic molecule and is based on mutually reinforcing feedback loops. ATc prevents inhibition of *lacI* by transcription of TetR. Transcription of the toxin is inhibited by LacI, preventing cell death. Furthermore, LacI weakly represses *tetR* and strongly represses transcription of the protease Mf-Lon, which degrades LacI. Using a combination of the restriction endonuclease *Eco*RI (the toxin) and the degradation of the essential protein MurC by Mf-Lon, the researchers were able to achieve escape frequencies below the limit of detection. **b** The *Passcode* kill switch is based on galactose, cellobiose, and IPTG as three independent ‘input signals’. Input A (galactose) inhibits GalR-LacI, while input B (cellobiose) inhibits CelR-LacI. Both GalR-LacI and CelR-LacI can bind the LacI-ScrR operator, thus inhibiting expression of LacI-ScrR. LacI-ScrR represses the expression of the toxin and can be inhibited by input C (IPTG). The cell only survives in presence of galactose and cellobiose and in absence of IPTG. Escape frequencies below 1 × 10^− 6^ were reached using *Eco*RI and Mf-Lon as toxins

More complex kill switches like *Deadman* and *Passcode* (Fig. 2) were recently developed [28]. Both systems are based on one or multiple synthetic molecules necessary for the cell’s survival. The *Deadman* switch is based on mutually reinforcing feedback loops and uses anhydrotetracycline (ATc) as the synthetic molecule, which prevents inhibition of *lacI* transcription by TetR. LacI inhibits transcription of the toxin, hence preventing cell death. Additionally, LacI weakly represses *tetR* and strongly represses transcription of the protease Mf-Lon, which degrades LacI. The group also studied the possibility to use Mf-Lon to degrade an essential protein and found that a combination of the restriction endonuclease *Eco*RI (the toxin) and the degradation of the essential protein MurC results in survival rates (Table 2) below the limit of detection. The *Passcode* switch uses three independent ‘input signals’ (galactose, cellobiose, IPTG). Input A (galactose) inhibits GalR-LacI, while input B (cellobiose) inhibits CelR-LacI. Both GalR-LacI and CelR-LacI can bind the LacI-ScrR operator, thus inhibiting expression of LacI-ScrR. Expression of the toxin is repressed by LacI-ScrR, which can be inhibited by input C (IPTG). Therefore, the cell only survives in presence of galactose and cellobiose and in absence of IPTG. Using again *Eco*RI and Mf-Lon as toxins, cell survival ratios below 1 × 10–6 were reached.

## Kill switch: DNA destruction

Kill switch systems for GMOs are widely used as biosafety systems. But, depending on the TA type (I to V) used, genetic information can potentially be released and spread through HGT [26, 32, 126, 127]. Therefore, genetic information has to be released, taken up by another organism, maintained and expressed [26]. If the uptake of this genetic information has an evolutionary advantage for the recipient cells, for example the information for an antibiotic resistance gene, it is likely to remain in the genome. Thus, genetic incorporation can lead to resistance to antibiotics or other advantages for recipient cells. Adapting a combination of toxin-antitoxin systems and self-destruction systems prevents the spread of recombinant DNA by the degradation of nucleic acids, while the targeted cells are killed. Therefore, self-destruction can be seen as a special case of a kill switch system. All systems based on destruction of nucleic acids can be loosely subdivided into three systems depending on the sequence specificity of used systems: (i) specific systems are based on nucleases which hydrolyze nucleic acids at sequence specific sites known as restriction sites, (ii) unspecific systems utilize nucleases which hydrolyze DNA and are not sequence specific and (iii) a third system which is a combination of sequence specific and unspecific parts.

Specific systems of self-destruction are toxin-antitoxin systems based on nucleases applied to kill cells with minor risk of DNA leakage. Usually, the nuclease (toxin) is encoded on the plasmid, while the nuclease-methyltransferase (antitoxin) is encoded on the chromosome. Both nuclease and nuclease-methyltransferase compete for the same sequence-specific DNA recognition site. Thus, expression levels between this toxin-antitoxin gene pairs need to be adjusted. The toxin is constitutively expressed and the expression of the antitoxin is induced, for instance, by using an anhydro-tetracyclin-responsive promoter [76]. When expressed the nuclease-methyltransferase methylates certain nucleobases at the recognition site, hence preventing cleavage of the DNA by the nuclease. This modification prevents digestion of the cell’s own DNA. A known toxin-antitoxin system is *Eco*RI/*Eco*RI methyltransferase [128–132]. Destruction of the genomic information is initiated by switching off the expression of the antitoxin-methyltransferase. Even if HGT occurs, the recipient cells are unlikely to counteract the nuclease due to the lack of methyltransferase.

The team University College London 2012 [133] used the *Eco*RI/*Eco*RI methyltransferase system in their novel threefold active biological containment system (Table 1) in combination with holin/anti-holin endolysin and colicin-E3/colicin Immunity E3 (BBa_K729009, BBa_K729010). The aim was to minimize HGT via bacterial conjugation using this new system.

At the end of the last century, genes encoding sequence specific restriction nucleases under different promoters were characterized for inducible degradation of DNA [134]. Stephen Cuskey performed preliminary tests with *Eco*RI under control of an inducible promoter as mentioned by Molin et al. 1993 [134]. Results showed that growth was reduced due to a high base level of gene expression without induction. Double strand breaks caused by *Eco*RI at a high rate are responsible for slow growth, as the repair mechanisms are insufficient to fix them [134].

Nonspecific nucleases, which introduce single strand breaks, combined with an inducible promoter, a ribosome binding-site, and a start codon, appeared to be better candidates [134, 135]. Molin and Ahrenholtz both tested the extracellular nuclease of *Serratia marcescens* [134, 135]. The *nucA* gene encodes a 266 amino acid long polypeptide with a 21 amino acid long N-terminal leader peptide [136, 137]. In both cases, the *nucA* gene without the leader peptide was under control of an inducible promoter *lac* and lambda *p*_*L*_, respectively [134, 135]. NucA is one example for an unspecific self-destruction system.

The team TU Munich 2013 [138] used the thermonuclease NucA of *Staphylococcus aureus* (BBa_K1159105) to degrade DNA of a genetically modified moss (Table 1). The nuclease (BBa_K1159111) is bound to the membrane with a transmembrane domain (BBa_K1159315) and contains a TEV cleavage site as well as a SV40 nuclear localization signal (NLS) (BBa_K1159303). After PhyB (BBa_K801031) is activated with red light, it binds either protein PIF3 (BBa_K1159103) or PIF6 (BBa_K1159104). This binding initiates the assembly of the N- and C-terminal split TEV protease which in turn cleaves the TEV cleavage site thus releasing the nuclease; this is translocated into the nucleus where the DNA is degraded.

Team NTU-LIHPAO-Taiwan 2015 [139] also used the thermonuclease NucA of *S. aureus* (BBa_K1159105) under control of a lambda cl-regulated promoter (BBa_R0051) to degrade DNA, thus killing *Lactobacillus casei* if the cl protein (BBa_C0051) concentration decreased below a certain threshold. The expression of *cl* is controlled by the *lac*-promoter and therefore regulated by lactose and glucose. The whole system is hypothetically designed to inhibit HGT from *L.casei* to bacteria in the human gastrointestinal tract while controlling the proliferation of the cells.

While sequence specific and unspecific systems may be of use in general self-destruction, the expression controls can be leaky leading to a baseline expression which is damaging to the cells even if they are contained. Also, both systems are not able to target specific sites which encode vital enzymes and proteins and are thus inefficient at mediating cell death. A combinatorial system of sequence specific and unspecific parts can be adapted for high-efficiency, easily controllable biosafety.

A self-destruction system which is based on sequence specific and unspecific parts is the clustered regularly interspaced short palindromic repeats (CRISPR)/CRISPR associated (Cas) system - the RNA-mediated adaptive defense systems of bacteria [140–144] Shortly after its discovery, it was adapted for genome and transcriptome editing [145–149]. The sequence-specific CRISPR is used to guide the sequence-unspecific Cas nuclease to its target, thus making this system highly regulated without the risk of uncontrollable cleavage. Employing the same mechanism on essential and non-essential genes enables controlled degradation to prevent HGT in the event of an unintentional release [150–152]. In nature three types of CRISPR/Cas systems are specified with variations concerning target and mechanism: type I systems cleave and degrade DNA; type II systems solely cleave DNA; and type III systems cleave DNA and RNA [153]. For the purpose of self-destruction, the type I system is well suited and frequently employed [151, 152]. Type I and type II systems are dependent on (i) CRISPR RNA spacer and target protospacer sequence complementarity as well as (ii) the protospacer-adjacent motif (PAM) [154–157]. By combining different Cas proteins and PAM sequences, a broad range of applications can be enabled influencing the kinetics of target degradation [151–153, 158]. Type III systems also require spacer-protospacer complementarity and specific sequences in the neighborhood of the protospacer [159]. Specific ON and OFF states of expression should be defined when employing the CRISPR/Cas mechanism for degradation of DNA to ensure induction of expression of both components in response to specific environmental changes [152].

Team HKU Hongkong 2015 [160] designed a CRISPR/Cas9 system with a specific sgRNA (BBa_K1774000) to target the DNA polymerase III alpha subunit (*dnaE*) thus inhibiting replication of the bacteria. The OFF state was defined by the availability of arabinose and tryptophan. Arabinose induced the expression of *araC* which in turn induces the expression of *cl* under control of the P_BAD_ promoter, thus inhibiting the P_R_ promoter and the expression of *cas9*. If available, tryptophan binds to a repressor which in turn blocks the *trp* promoter and thus sgRNA expression. In the ON state, arabinose and tryptophan are not available, which mimics a possible physical containment breach. A lack of transcriptional repression results in the formation of *Cas9* and the sgRNA, which can destroy the gene of DNA polymerase III alpha subunit.

Given that kill switches are prone to inactivating point mutations [161], especially when constitutively expressed, researchers have developed new biosafety systems that do not harm the host, potentially minimizing the risk of unintended proliferation caused by mutagenesis. Jia and colleagues developed an orthogonal ribosome biofirewall, consisting of an activation circuit and a degradation circuit [162]. The activation circuit, a genetic AND gate, utilizes an orthogonal ribosome to activate an encrypted pathway based on specific environmental inputs. The genes encoding the orthogonal ribosome can be degraded by the degradation circuit, a genetic NOT gate, based on a change of the environmental inputs. This elegant system not only minimizes the burden on the host, given that the toxin I-SceI is not constitutively expressed, but also makes expression of the genes of interest dependent of the presence of the orthogonal ribosome. Therefore, even if HGT occurs, expression of the genes of interest is prevent due to the lack of an orthogonal ribosome. The plasmid containing the genes for the orthogonal ribosome is digested in absence of specific environmental inputs. This work highlights how conditional degradation of genetic information can be combined with genetic encryption to create an adaptable and tightly regulated biofirewall for microbial biocontainment [162].

### Xenobiology-based containment

The term ‘xenobiology’ has experienced significant semantic shift over the last few decades [163–165]. Kubyshkin et al. define xenobiology as an approach to expand the framework of natural chemistries with non-natural building blocks in living cells to accomplish artificial biodiversity [50]. Therefore, one key aspect of xenobiology is the search for alternative chemistry for nucleic acids, proteins and other cellular components and functions. Xenobiological systems are also referred to as orthogonal systems or chemically modified organisms (CMOs) [50, 163]. Current biosafety systems are meant to kill cells once they escape from the assigned environment, leaving their recombinant DNA freely available in nature [166]. An orthogonal system prevents HGT [167] through transduction [168], conjugation, and transformation, as reviewed by Davison [169]. As a consequence, wild type cells are unable to integrate and maintain XNA into their genome and cannot handle the incorporation of ncAAs [170, 171]. Thus, XNA can potentially become a powerful biosafety tool by preventing HGT as it should not be read properly by wild type DNA processing enzymes like DNA and RNA polymerases [164].

The classification of xenobiology is not consistent. Some categorize it along with trophic and semantic containment for the prevention of metabolic and genetic exchange [171]. Giving this inconsistency, the following chapters are categorized according to the components that could be changed or alienated like the bases, backbone, leaving group, codons and amino acids.

#### Synthetic bases – building up Xeno-DNA

During the emergence of recombinant DNA technology, a plasmid containing DNA of another species was described as Xeno-DNA (XNA) [20]; the modern perception of XNA describes non-canonical DNA building blocks or substantial modifications of the natural structure, such as alternative pairing nucleotides, modified sugars, or backbones [165, 172, 173]. XNA could be considered a genetic firewall masking the encoded information from nature [163]. The main approach of designing XNA is to replace or extend the standard genetic code comprising four naturally occurring nucleotides in the DNA. There are various sophisticated approaches to identify potential replacements for the four canonical bases [174–181]. Nevertheless, advances in XNA technology have to fulfill some requirements to establish stable products in vivo [172, 182].

First experiments extended the four nucleotide alphabet by replacing thymine with 5-chlorouracil in *E. coli* over a period of 25 weeks [183, 184]. Other approaches expanded the genetic alphabet by introducing the two artificial bases dP (2-amino-8-(1′-β-D-2′-deoxyribofuranosyl)-imidazol[1,2-α]-1,3,5-trizan-4(H)-one), and dZ (6-amino-5-nitro-3-(1′-β-D-2′-deoxyribofuranosyl)-2(1H)-pyridone) [185]. These artificial bases pair with three hydrogen bonds but vary in the pattern of donor and acceptor groups. A Taq DNA polymerase was modified to accept the new ATCGPZ-DNA, resulting in a retention rate of 98.9% [186, 187]. Moreover, a T7 RNA polymerase and a reverse transcriptase were developed for an RNA product containing P and Z [188]. The six nucleotide genetic alphabet will lead to DNA with a B-form as well as an A-form, with the major groves being 1 Å wider than the natural G:C pair [189].

Interestingly, the concept of DNA can be extended beyond bases and pairing through hydrogen bonds. For example, pairings dependent on metal ion coordination [190–192] or hydrophobic interactions [193] were explored recently. Two promising candidates using hydrophobic interactions are d5SICS – dMMO2 and d5SICS-dNaM, which allowed transcription [193]. The first demonstration in *E. coli* was based on one plasmid encoding the nucleoside triphosphate transporter for dNaM and d5SICS and the other plasmid encoding a gene sequence using the extended genetic code [194]. Uptake of the synthetic bases as well as a stable plasmid replication over 24 generations was demonstrated [194]. In 2017, the Romesberg group presented a new version of their semi-synthetic organism. The most important advances were an optimized transporter with improved uptake of unnatural triphosphates and better retention of XNA with dNaM-dTPT3. Furthermore, they used a CRISPR-Cas system to eliminate plasmids that lost the XNA [177].

Besides expanding the alphabet of canonical DNA, XNA provides the opportunity to change the general topology. For example, benzo homologation provides the opportunity to expand the physical DNA size. The benzo expansion of pyrimidines to create dxT and dxC results in expanded DNA (xDNA), with the size increasing about 2.4 Å and the helix becoming more thermally stable [195, 196]. Stable replication of a plasmid containing up to eight xDNA bases in a GFP encoding sequence as well as expression of the altered *gfp* gene was demonstrated in *E. coli* [197]. By further changing the vector of extension, wide DNA (yDNA) can be obtained [198], although stable replication of this DNA type is problematic [199].

Use of XNA often necessitates synthetic or evolved proteins that allow for replication, transcription, and DNA packaging of the XNA. All presented examples depend on supplementation of the non-canonical nucleotides. This auxotrophy is a potent biosafety mechanism, which does not just prevent an uncontrolled growth of the engineered cells in the environment, but also protects the encoded information from spreading through HGT [163, 171].

#### Alternative XNAs: modifying backbone or leaving group

Besides the incorporation of non-canonical bases, experiments to engineer the DNA backbone by integrating substitutes for deoxyribose and ribose have been performed. For biosafety purposes, an altered backbone needs to meet the requirement to build a functional helix that does not interact with natural replication enzymes, instead requiring adjusted or even synthetic enzymes [172]. Some candidates for alternative backbone chemistries have previously been investigated as reviewed by Herdewijn and Marlière [172]; these examples include: hexitol nucleic acid (HNA) [200], threose (TNA) [201, 202], glycerol (GNA) [203], and cyclohexene (CeNA) [204]. A less complex method compared to the substitution of the whole genomic backbone is utilizing an orthogonal XNA episome which contains essential genes [172].

Besides the bases and the backbone, the third potential target to design XNA is alternating the leaving group of NTPs by replacing the pyrophosphate, such that they cannot be recognized by wild type polymerases. Studied analogues of ATP are methylene phosphonate, phosphoamidate [205], and thiophosphonate [206]. An alternative leaving group needs a high energy bond for the polymerization process. Studies on L-aspartate and L-histidine linked nucleotidemonophosphates showed that aspartic acid phosphoramidate derivates are working substrates for the HIV reverse transcriptase [207].

#### Amber codon and non-canonical amino acids

By systematically expanding the approach of auxotrophies based on xenobiotic compounds to utilizing a whole orthogonal genetic code, e.g. by means of xenobiology, the spread of recombinant sequences can also be prevented. There are three stop codons in the genetic code: ochre (UAA), opal (UGA) and amber (UAG) [208]. In *E. coli*, the amber codon is least common, with just over 300 occurrences (depending on the *E. coli* strain used). In 2013, Lajoie and colleagues coined the term genetically recoded organism (GRO) to describe organisms with an alternative genetic code [209]. Such GROs have been developed for enhanced biosafety compared to natural amino acid auxotrophs by engineering organisms to become auxotrophic for non-canonical amino acids (ncAAs) [58]. Rovner et al. constructed GROs based on *E. coli* without any TAG codon and the possibility to terminate translation at the UAA and UAG codons. After recoding the TAG codon to a sense codon for ncAAs by means of an orthogonal translation system (OTS), the recoded codon was incorporated into essential genes of the organism, thus making it dependent on ncAAs. Sixty variants of the auxotroph-GROs with varying growth and containment rates have been isolated, with one strain containing three recoded TAG codons maintaining stable growth, and undetectable escape frequencies over the course of 1 week or 20 days on solid or in liquid media, respectively. Changing the amber codon into a sense codon has been done multiple times by various groups [210–217], but results in the mistranslation of all genes using this stop codon. Therefore, the Church lab presented a GRO in which all 314 UAG stop codons were replaced by UAA stop codons. Deletion of release factor 1 (encoded by *prfA*), which recognizes UAG and UAA, then allows for recoding of the amber stop codon [218, 219]. Engineering a new aminoacyl-tRNA synthetase (aaRS) and corresponding tRNA leads to an orthogonal translation machinery required to harness the potential of an amber-free strain [220]. The non-canonical amino acid (ncAA) L-4,4′-biphenylalanine (BPA) had been integrated via the UAG stop codon of the GRO *E. coli* strain C321.∆A by Mandell et al.. This resulted in auxotrophs designed to be dependent on ncAAs [57]. Thus, the essential enzymes of the strain required BPA for core functions, such as translation. Additionally, in a proof-of-concept study, an *E. coli* strain BL21-AI (IY, *lamB*-*immE3*) containing the synthetic essential gene *immE3* had been constructed, the translation of which depends on supplementation of the medium with the non-canonical amino acid 3-iodo-L-tyrosine [221]. As an alternative to reassigning stop codons, it is also possible to reassign sense codons to ncAAs. In order to ensure proper functioning of the altered strain, rarely-used sense codons have been used, such as the codon AGG, which usually codes for arginine and has been reassigned to code for the ncAA L-homoarginine in *E. coli* [222]. Such an approach has been explored with other sense codons, such as AUG [223], and as a combination of sense and stop codon reassignment [224]. Reassigning single codons is not the only way to incorporate ncAAs to the genetic code. Instead, Hoesl and colleagues [225] have evolved cultures of *E. coli* to grow on a non-canonical amino acid alternative to L-tryptophan (L-β-(thieno[3,2-b]pyrrolyl)alanine) in a long-term cultivation experiment. While cells were capable of surviving in the total absence of L-tryptophan, they were still able to grow when L-tryptophan was present. In the future, further strain engineering might provide an evolutionary approach to altered strains dependent on ncAAs.

The resulting combination of trophic and semantic containment constitutes a powerful biosafety system. While not preventing the transfer of genetic material in the first place, the recoded DNA cannot be expressed after an HGT event in natural organisms. Moreover, HGT of sequences encoding an aaRS and the corresponding tRNA are either lethal or very detrimental in natural, non-recoded organisms as they will lead to mistranslation of amber containing genes.

In 2006, Wang et al. recoded the amber codon to implement a non-canonical amino acid. The source organism of the altered tRNA and aminoacyl tRNA synthetase for tyrosine was *Methanococcus jannaschii* [226]. After engineering, this system was able to insert O-methyl-L-tyrosine in a gene encoding the dihydrofolate reductase. Another example, based on a translation switch controlled by the absence of 3-iodo-L-tyrosine [221, 227], is described in the section on auxotrophy-based systems.

Shifty codes [228] are used to encode the same product as expected in the wild type, but are based on quadruplets and orthogonal ribosomes. The evolution of orthogonal ribosomes translating a quadruplet code provides the amazing opportunity to assign 256 blank codons. Neumann et al. [229] evolved a synthetic ribosome (ribo-Q1), whose decoding fidelity was as high as in wild type ribosomes [230]. They tested the incorporation of two non-canonical amino acids, p-azido-L-phenylalanine (AzPhe) and N6-[(2-propinyloxy)carbonyl]-L-lysine) (CAK), encoded by a combination of a quadruplet codon AGGA and the amber codon UAG in *E. coli*. The protein was only completely synthesized when both non-canonical amino acids were encoded in the DNA [229, 231, 232].

#### Application of xenobiology-based containment in the iGEM competition

In 2012, team Paris Bettencourt [104] worked on an extensive biosafety project (Table 1). A semantic containment part was based on an amber mutation in the gene conferring kanamycin resistance (BBa_P1003). The objective was to prevent expression of the antibiotic resistance gene in wild type bacteria cells after a HGT event. Two parts were constructed to realize the amber suppression in *E. coli* MG1655: BBa_K914000 encoding P_*lac*_-*supD*-T: tRNA amber suppressor and BBa_K914009: P1003^*Ser133^ encoding a kanamycin resistance gene with one amber mutation at a serine residue at position 133.

The team demonstrated that the amber codon was effectively recoded. The growth rate and level of resistance were not significantly decreased compared to the strain carrying the original kanamycin resistance gene as well as the tRNA amber suppressor. However, the culture without the tRNA amber suppressor reached a higher OD_600_ value, because other amber stop codons on the chromosome were also suppressed. Interestingly, they found that a single amber mutation was quickly overcome by mutations, a problem that could be addressed by introducing a second amber mutation in the kanamycin resistance gene. Thus, amber codons within antibiotic resistances are an effective way to prevent the easy spread of such resistances. However, further improvements of the system are needed to prevent the HGT of the amber suppressing tRNA.

The team TU Darmstadt 2016 [233] combined auxotrophic incorporation of a non-canonical amino acid and a reporter for low levels of the ncAA (BBa_K1416000, BBa_K1976025) [234] designed by the team Austin Texas 2014 [235]. Amber codons were placed at the beginning of a Colicin E2 immunity protein [236] and the mutated Zif23-GCN4 repressor (F4OMT), a dimeric Cis2His2 zinc finger protein [237]. In case of the absence of the ncAA, both proteins cannot be translated, resulting in expression of the reporter system mVenus [238] under control of a Zif23-GCN4-controlled promoter and subsequent initiation of the suicide reaction. However, no results were reported on the expression of the reporter and the OMT-RS expression under a T7 promoter (BBa_K525998).

Team Bielefeld-CeBiTec 2017 worked on expanding the genetic code with the unnatural base pair formed between isoguanosine (isoG) and 5-methyl-isocytosine (isoC^m^), and non-canonical amino acids [239]. The team used CRISPR/Cas9 to retain the unnatural base pair in a specified sequence. Furthermore, the algae transporter *Pt*NTT2 was used to facilitate uptake of the unnatural nucleoside triphosphates. Given that this transporter can also facilitate transport of ATP, it may also be used to engineer an artificial ATP auxotrophy. By recoding the amber codon, the team incorporated ncAAs in a number of proteins. Cultivations showed growth defects if the desired ncAA was not supplemented. The cells were still able to grow as the aaRS, with lower affinity, also incorporated endogenous amino acids. Therefore, engineering artificial ncAA auxotrophies requires highly specific aaRS.

## Minimal genome

Sometimes referred to as the “holy grail” of synthetic biology [240–243] the minimal genome is defined as a set of genes which are essential for survival of the cell [244] in an environment containing all required supplements for life [240]. The size of a minimal genome depends on the surrounding environment [245]. Therefore, the sets of required genes differ slightly as reviewed by Gil and colleagues [246]. Most approaches rely on transposon mutagenesis [247] or antisense RNA [248, 249] to identify essential genes [250]. Nevertheless, it is hard to determine the minimal set of essential genes, since an essential function might be encoded by two or more genes, thus resulting in false negative assignments [245, 251].

Minimal genomes can be constructed via “top down” or “bottom up” approaches, with “top down” being the systematic deletion of redundant genes, while “bottom up” describes the synthesis and assembly of a genome with the minimal set of genes [252]. Numerous, sophisticated attempts were made to identify essential genes of an organism in order to construct a minimal genome of *Haemophilus influenzae* [253], *H. influenza* and *Streptococcus pneumoniae* [254], *Mycoplasma genitalium* [255, 256] *S. aureus* [249], *Buchnera spp*. [257], *Saccharomyces cerevisiae* [258], C*orynebacterium glutamicum* [259] and *E. coli* [260–262].

In *M. genitalium*, which had the smallest known genome at that time [263], global mutagenesis led to the identification of 265 essential genes [256] which aligns well with the published predicted minimal genome consisting of 256 genes [264]. An 1.08 Mbp synthetic genome sequence of *M. mycoides* was assembled and transplanted into an *M. capricolum* recipient cell producing *M. mycoides* JCVI-syn1.0 [265]. Latest research has led to the development of a minimal genome of *M. mycoides* called JCVI-syn3.0 [245]. Based on JCVI-syn1.0, the genome reduction was achieved in 2016 in the new synthetic 532 kbp genome of JCVI-syn3.0 [245]. It contains the minimal set of essential genes, although the function of 149 genes remained unknown. Further reduction processes identified quasi essential genes, which were not required for survival, but contributed significantly to stable growth [245]. Cells with a reduced genome showed a duplication time of 180 min, which is substantially different to the duplication time of 60 min of JCVI-syn1.0 with a gene set for robust growth [245].

A bacterial cell with a minimal genome offers various applications in biosafety. The cells would depend on complex media as well as on stable conditions. Therefore, survival in a natural environment with fluctuations in environmental conditions is improbable. However, HGT is still an issue in this scenario, which is why we recommend aiming for a combination with self-destruction systems as described above.

Since the iGEM competition is about the construction of plasmid-based BioBricks, the development of a minimal genome would be challenging for the teams. Still, teams like Alberta 2009 [266], ETH Zurich 2008 [267], and UESTC 2015 [268] developed concepts and implemented software to identify minimal gene sets based on bacterial genome sequences.

## Cell-free systems

The main concerns from a biosafety perspective are the possible release of GMOs into the environment and HGT between engineered and wild type organisms. Cell-free protein synthesis (CFPS) represents a promising possibility to eliminate most of these risks. The first researchers to show that disrupted bacterial cells can be used for in vitro protein synthesis were Gale and Folkes in 1954 [269]. In 1961, Nirenberg and Matthaei conducted pioneering work using *E. coli* cells and showed that template RNA is a requirement for cell-free protein synthesis [270]. CFPS is feasible for a variety of applications, including synthetic biology, vaccine production and protein engineering [271–273].

There are two main strategies for CFPS. The first, older one is based on crude cell extracts from the desired cells. While the necessary crude extracts are easy to prepare, fast energy depletion and degradation by proteases and nucleases pose two major problems [274–276]. To counter those problems, the PURE (“protein synthesis using recombinant elements”) system developed by Shimizu et al. 2001 [276] can be used. This cell-free system is based on purified (His)-tagged translation factors and can be programmed by natural mRNA. For biosafety reasons, it is important to effectively remove all living cells before deploying a CFPS system outside of the lab. While standard methods for the preparation of cell-extracts are already highly effective, they still do not provide a completely cell-free extract [277, 278]. Protocols based on sterile filtration and lyophilization can provide a much more sterile extract, minimizing the risk of accidental release of genetically modified organisms into the environment [278]. To fully circumvent the risk of an unsterile cell-extract, the aforementioned PURE system can be deployed. This system is not only safer, but also has lower energy consumption relative to S30 cell extract systems (cell extracts cleared from heavier components by centrifugation at 30,000 xg), with greater productivity. However, Shimizu et al. could not top the productivity of 400 μg/ml with a S30 extract when they first published their results [276, 279].

Several iGEM teams used cell-free systems for their projects. Bielefeld-CeBiTec 2015 (Table 1) used cell extracts of *E. coli* KRX and ER2566 strains to produce sfGFP as part of a paper-based biosensor [280]. Both strains feature low endogenous protease activity and a chromosomally integrated T7 polymerase. To create a cell extract, the team harvested the cells at mid-to-late exponential growth phase and sonicated the cells as described by Kwon and Jewett [281]. Using this system, the team successfully produced sfGFP in vitro on a paper strip.

Teams Edinburgh 2015 and Exeter 2015 also tried to build a biosensor using cell-free protein synthesis. Edinburgh used *E. coli* BL21 to express the desired enzymes fused to cellulose-binding domains. The cells were freeze-dried to obtain an extract containing the enzymes which were then immobilized on paper to create a paper-based drug testing biosensor [282]. Exeter 2015 developed a biosensor for the detection of bovine tuberculosis. The team used a commercially available S30 cell-free kit to express GreenFET1J as a response to the trigger RNA [283].

Team Freiburg 2015 tried to build a microchip for simultaneous detection of several infectious diseases [284]. The team used an *E. coli* lysate in a microfluidic chamber and expressed HA- and (His)-tagged GFP as well as luciferase as a proof of concept. Their final goal was to express disease-specific antigens like the *Clostridium tetani* antigen, however even after optimization, antigen production could not be detected.

In 2017, team Lethbridge worked on a standardized, modular system for CFPS with their project ‘next *vivo*’ [285]. The design of the system was based on standardized expression and purification of all proteins required for transcription and translation, the ribosomes, as well as the necessary tRNAs. The team aimed to overexpress all 38 essential proteins required for transcription and translation in *E. coli* BL21-Gold (DE3) to subsequently pool and co-purify all components. The team successfully overexpressed and purified key proteins required for CFPS and succeeded in purifying tRNA^Phe^ as a proof of concept. Additionally, the team hypothesized the use of a modified codon table as a biocontainment strategy for CFPS.

## Physical containment

While biological strategies have strengthened biocontainment, most strategies, especially in industry, are centered around physical containment [56]. We define physical containment as the separation of cells from the environment by means of physical materials e.g. the wall of a bioreactor [23]. Physical containment acts as a preemptive strategy intended to prevent the release of GMOs and includes the design of equipment as well as facilities used [56]. Besides “classical” full containment systems like bioreactors, cell retainment systems which allow the exchange of small molecules, e.g. micro encapsulation, enable a broad range of applications [286–289]. In theory, cells should secrete their product, e.g. neurotransmitter [290, 291], continuously over a long-time period. However, this mechanism requires expensive viability controls and the development of mechanisms to prevent unintended release of bacteria from capsules [292]. Stability of encapsulation can be increased by chelating compounds, anti-gelling cations like Na^+^ and Mg^2+^ or polymers [293, 294].

The survival rate of cells in silica gels as encapsulating substance [295–297] reached 55% living cells after 4 weeks by adding glycerol as osmotic stabilizer [298]. The specific advantage of silica gels is their action as physical barriers between the cells, preventing cell aggregation and thus physical interaction between cells [298]. Although there is a mechanical and chemical stability of silica gels which ensure entrapment of cells, silica gels are still not as stable as polymer gels [299–301].

Since iGEM applications are often targeting environmental problems or offer applications for daily use, the development of solutions for encapsulated cells is a pressing need. Such physical containment strategies were applied by Paris Saclay 2015 to embed bacteria in silica beads [302]. They developed a novel protocol based on two publications [303, 304] which remains to be tested.

Paris Bettencourt 2012 worked on alginate beads for application with *E. coli*, based on a study with *S. cerevisiae* [305], using glutaraldehyde as a cross-linker [104]. This compound is toxic to unprotected cells but can be used together with polyethyleneimine to stabilize the cells within the alginate beads. Blue white staining was applied to demonstrate the viability of encapsulated *E. coli* cells. The stabilized beads showed a 1000-fold reduced cell-release rate. This was demonstrated by cultivating cells entrapped by both types of beads for 24 h followed by streaking out the supernatant on agar plates to quantify the number of released cells.

## Sensors

In a broader sense, physical containment can be understood also as the containment by physical parameters like temperature or light. These parameters are suitable as they are usually well controlled in a bioreactor. In addition, a number of control systems can be found in many organisms, as living cells must adapt to changes in the temperature and light condition of their environment. By sensing these (or other) environmental conditions, release of bacterial cells from a controlled environment can be linked to other biosafety systems, like kill switches.

### Temperature

Depending on the organism, release from the physical containment might result in either a heat or cold shock which could be used to differentiate between growth in a temperature-controlled environment like a bioreactor or in a natural environment, respectively [306, 307].

As many organisms encounter (sometimes drastic) changes in temperature in nature, a wide variety of sensors and response systems have evolved in bacteria and other organisms. For example, over 100 proteins were previously described to be involved in the heat shock response of *E. coli* [308–310]. Most of these proteins function as molecular chaperons or proteases, which preserve the protein structures, membrane homeostasis or nucleic acid topology [308–310]. On the other hand, cold shock proteins (CSPs) allow to tolerate low temperatures by maintaining efficient translation of RNA and membrane fluidity [311, 312]. Another group of proteins are methyl-accepting chemotaxis proteins (MCPs) which are involved in temperature-dependent changes in the movement of *E. coli*. Of these Tsr, Tar and Trg were described to detect heat shock temperatures while Tap detected low temperatures [313–316].

A more direct level of temperature-based control is realized on the DNA and RNA levels. For example, transcription efficiency is dependent on the DNA topology, especially on the level of supercoiling [317–319] which can increase or decrease due to temperature stress [320]. Another common control mechanism that has been recognized is on the level of RNA in the form of riboswitches. These elements are usually located within the 5′-UTR of protein encoding genes [321] and control protein biosynthesis through temperature-mediated structural changes [322–324]. They usually consist of temperature unstable hairpin loops [325], but more sophisticated RNA structures like the pseudo knot structure of the *cspA* transcript [326] are also known. The typical mode of action of these ‘RNA thermometers’ is sequestration of the RBS by hairpin formation which is released at elevated temperatures [327]. Beside cis-acting RNA molecules, at least one trans-acting RNA has been described: DsrA affects the expression of other genes through modulation of translation of *rpoS*, encoding the non-essential sigma factor of RNA polymerase in *E. coli*, at low temperatures by binding to the *rpoS* transcript, releasing the otherwise sequestered RBS [328–330].

Promising work has been done on the integration of riboswitches into biosafety systems regarding gene expression [331, 332]. Dedicated non-coding RNA molecules were frequently applied to measure temperatures in vivo [333–335]. Multiple examples of temperature sensing RNA molecules integrated as key part of a biosafety system were presented by iGEM teams during the last years (Table 1) [30, 31]. Team Paris Saclay 2015 applied the RNA-based temperature sensor technology to set upper (42 °C) and lower (32 °C) temperature boundaries for growth of *E. coli* [302]. The RNA molecule ROSE [336] from *Bradyrhizobium japonicum* [337] was proposed to control the expression of three essential genes in *E. coli*. Genes encoding the tRNA synthetases for alanine, tyrosine and methionine as well as the DNA polymerase III subunit delta might be good targets for conditional expression [262]. Other potential targets, which are essential, non-redundant and constitutively expressed, include adenylate kinase (*adk*) [338], alanyl-tRNA synthethase [339], DNA polymerase III subunit delta (*holB*) [340], methionyl-tRNA synthetase (*metG*) [341], phosphoglycerate kinase (*pgk*) [342], and tyrosyl-tRNA synthetase (*tyrS*) [343].

The native promoter sequences of three essential genes were substituted with a repressor-controlled *tet* promoter (P_*tet*_) [344]. ROSE was applied to control the expression of the TetR repressor to indirectly regulate the expression of all three tRNA synthetases. Since ROSE inhibits translation of the controlled mRNA below 30 °C [336], *E. coli* is only able to grow above this temperature when the Tet repressor is translated. The *cI* gene from bacteriophage λ was applied to achieve an upper growth limit, which may be important for applications involving thermotolerant or thermophilic bacteria. While an upper restrictive temperature is not necessary for application in most environments, the concept of producing a stenoecious (insensitive to environmental factors) organism by synthetic biology is very interesting.

Another good example is the use of the RNA molecule FourU from *Salmonella entericaserovar* Typhimurium M556 [345] (BBa_K115002) by the iGEM teams TU Delft 2008 [346] as well as NCTU Formosa 2011 [347]. Expression of essential genes is controlled by this temperature-sensing RNA molecule, permitting the *E. coli* cells to grow only at 37 °C. Growth at lower temperature is inhibited by the formation of a stable RNA hairpin structure which prevents the translation of essential genes [345]. Growth at higher temperatures is not possible, since the high temperature is detrimental to *E. coli*. Functionality of this part was demonstrated via *Renilla reniformis* luficerase [348] and GFP [349] respectively, serving as reporter genes.

### Light-regulated gene expression

Light exposure is a major difference between the controlled environment within a bioreactor and the natural environment. Therefore, light sensors [350, 351] might be applicable in microbial biosafety systems when coupled to kill switches or other previously described systems.

One way for perception of light in bacteria are LOV photoreceptors [352] based on flavine nucleotides as chromophores [353]. A synthetic biology approach replaces the oxygen-sensing module of the histidine kinase FixL [354] of *B. japonicum* [355] with the LOV photo sensor module of YtvA from *B. subtilis* [356]. The resulting histidine kinase YF1 can be inhibited by blue light. The combination of the response regulator FixJ of *B. japonicum* [357] and YF1 forms a synthetic two component system. This system controls the FixK2 promoter [355] allowing light-dependent repression of genes under P_*fixK2*_ control [352], realized on the plasmid pDusk [358, 359]. For biosafety purposes, the opposite behavior is usually desired, requiring the use of an inverter. A ready to use system is available via the plasmid pDawn which uses a Yf1-FixJ-downregulated *cI* as well as a cI-repressed P_R_ upstream of the gene(s) of interest [358, 359]. Despite the availability of BioBricks, only a few iGEM teams have proposed to use this system for biosafety. To our knowledge, no team has so far successfully used the system for this purpose.

Phytochromes are another type of a membrane-bound extracellular sensor for light detection which regulate transcription via intracellular response regulators [360]. A synthetic biology approach involves the fusion of this photoreceptor and an intracellular *E. coli* histidine kinase domain [361]. This system is based on the EnvZ-OmpR two component system of *E. coli* which is involved in porin expression upon osmotic shock [362]. The chimera protein was constructed by replacing EnvZ with the phytochrome Cph1 of *Synechocystis* sp. PCC 6803 [363, 364]. The light-sensing receptor part (phycocyanobilin) is not produced in *E. coli* wildtype. However, phycocyanobilin biosynthesis can be achieved in *E. coli* by introducing the genes *ho1* (BBa_I15008) und *pcyA* (BBa_I15009) of *Synechocystis* sp. PCC6803 resulting in conversion of haem into phycocyanobilin [365, 366]. The chimeric protein Cph8 (encoded by BBa_I15010) reacts to light input with a strong response. This systems allows expression of the target gene(s) in the dark to be inactivated by red light [361].

The teams of Uppsala 2011 [367] and Cornell 2011 [368] conceptualized light-dependent biosafety systems. Green light was used by the Cornell team to trigger the expression of a lysis cassette. As green light is absent under normal cultivation conditions, this system should kill all cells outside a bioreactor. Cornell 2011 planned the combination of *ccaS* and *ccaR* on one plasmid in combination with the phycocyaobilin biosynthesis genes *ho1* and *pcyA* (BBa_K597105).

## Conclusion

We reviewed a broad spectrum of biosafety mechanisms and have highlighted promising achievements within the iGEM competition (Table 1). Auxotrophies, kill switches, mechanisms for self-destruction, and semantic as well as physical containment are important categories of orthogonal biosafety systems. Moreover, cell-free approaches provide biosafety to synthetic biology by avoiding living cells.

**Fig. 3:**
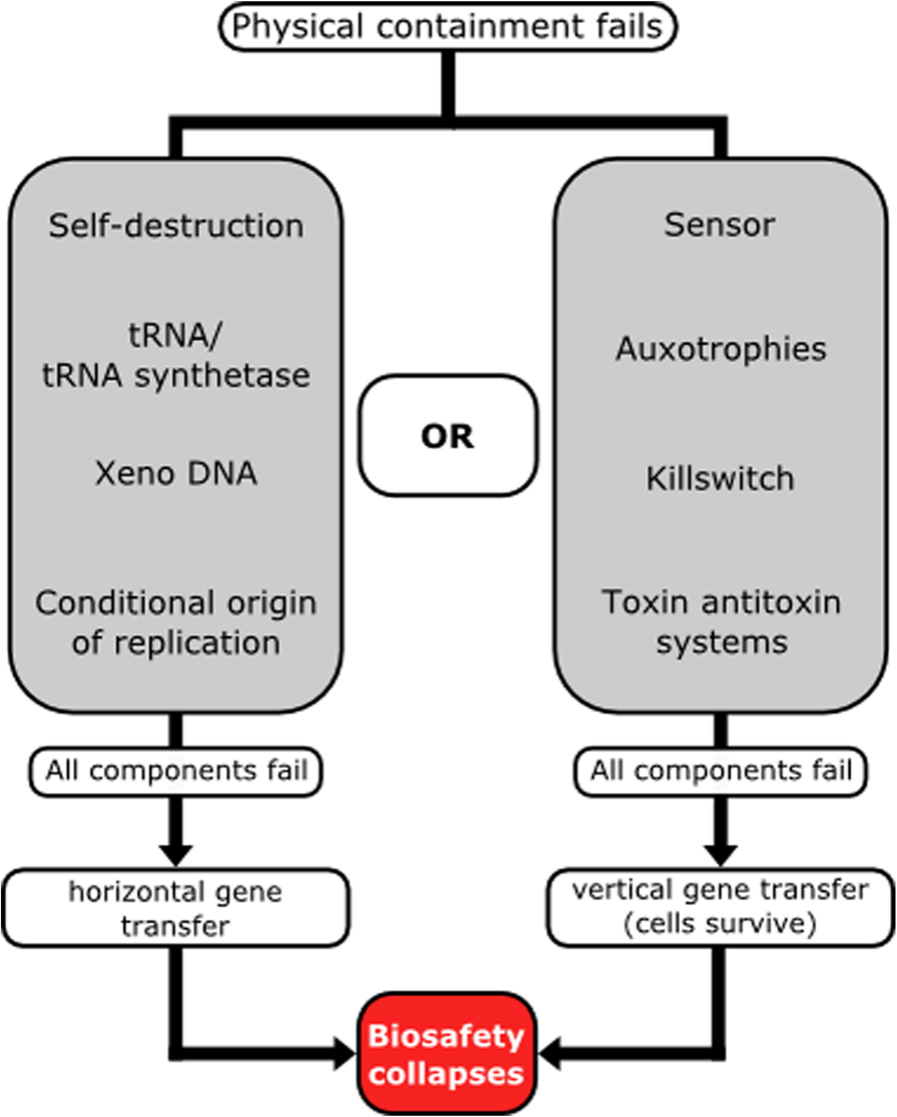
Biosafety defense level. Assuming that physical containment fails, and the engineered bacteria escape their designated environment (e.g. a bioreactor), there are two ways possibly leading to a total biosafety system collapse. A biosafety collapse happens once either HGT or VGT (reproduction) occurs. Therefore, transfer of genetic information from the engineered cell to a wildtype cell can only occur if all mechanisms preventing either HGT or VGT, respectively, fail

**Fig. 4:**
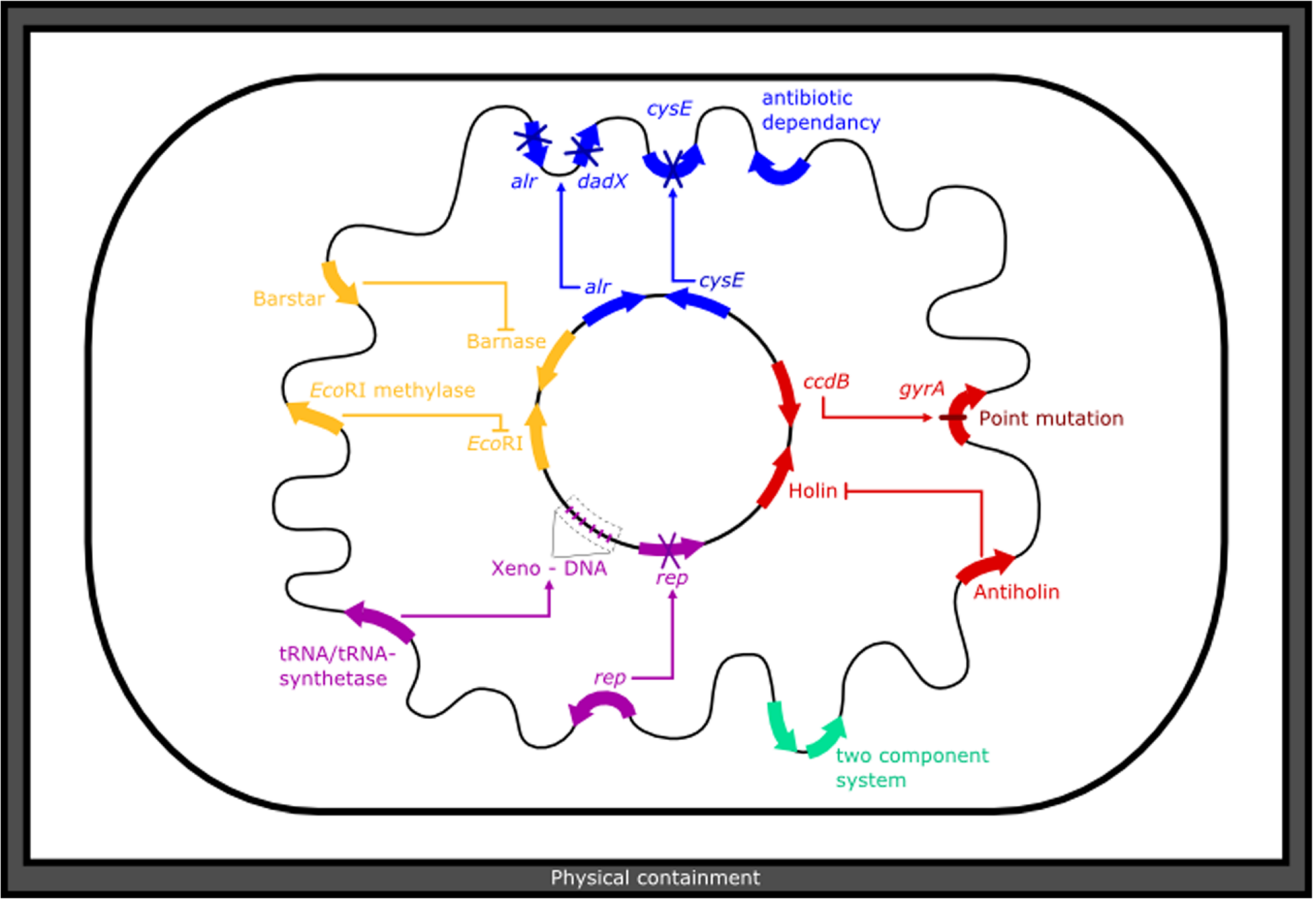
Comprehensive combination of biosafety mechanisms in *E. coli*. Proposed combination of orthologous biosafety mechanisms. Auxotrophies (blue), TA-systems (red), replication control mechanisms (purple) and self-destruction systems (yellow) could be combined to achieve a high-quality biosafety system. Furthermore, the proposed combination of systems includes physical containment (grey) and a two-component system (green) to enhance the reliability even further. To create artificial auxotrophies, *alr*, *dadX* and *cysE* were deleted in the genome and must be replaced with plasmid-bound gene copies. CcdB and Holin serve as toxins, but their toxicity will only effect wildtype cells. The toxicity of CcdB can be avoided through a single point mutation within the *gyrA* gene. To neutralize the toxicity of holin, an antiholin-encoding gene is present in the genome of the desired host. By moving the *rep* gene from the plasmid to the genome, the plasmid can only replicate if Rep is provided *in trans*. Incorporation of artificial bases into the plasmid (Xeno-DNA) prevents wildtype cells without the corresponding tRNA/tRNA-synthetase to produce any of the encoded proteins. To destroy the plasmid DNA if taken up by wildtype cells, self-destruction systems like barnase and *Eco*RI are included. Only the desired host possesses the corresponding inhibitors Barstar and *Eco*RI methylase and hence can counteract the toxicity

Despite these sophisticated mechanisms, responsible and careful handling of genetically engineered bacteria is the first step and most efficient way to ensure biosafety. However, in the case of an accidental release of genetically engineered bacteria, several mechanisms should be in place to prevent the spread of the engineered cells or their genetic information. It is important to emphasize that genetic components, process conditions and manufacturing design can all contribute to biocontainment and should therefore be taken into consideration to further increase the degree of biosafety [56]. Most standard safety strains are only based on auxotrophies, designed to prohibit growth in absence of one specific compound. Even if genetically modified cells are not able to survive in the environment, the presence of recombinant genetic information still provides opportunities for HGT. As any single biosafety system might fail with roughly the probability of a point mutation (10^− 7^) [369, 370], a combination of multiple systems can increase the safety in a linear way (Fig. 3). Therefore, it is important to combine orthogonal types of biosafety mechanisms within one cell [32, 76], resulting in safer, more sophisticated, synthetic biosafety systems. Such systems can be combined within one cell, as highlighted in (Fig. 4).

Calculating the worse-case probability, we assume only a linear safety increase which would require five different systems to reach a failure probability of 10^− 35^. Since recent estimations assume the presence of only about 10^30^ cells on earth [371], one could consider such a combined system as sufficiently safe for a single application. To achieve a high level of biosafety, several systems preventing HGT as well as VGT should be combined as each system might fail with a certain probability (Fig. 3, Table 2). However, it is important to keep in mind that the number of synthetic biology applications will increase significantly in the near future. Like the correction for multiple testing in statistics, the failure probability of biosafety systems in the future should be adjusted in accordance to the number of parallel applications. Therefore, a general awareness of potential risks associated with this technology is crucial. Whilst the number of early stage researchers utilizing methods of synthetic biology has increased through the iGEM competition, the teams have regularly conceptualized and successfully implemented novel biosafety systems (Table 1). The awareness for this topic seems to be higher than in general research. A dedicated biosafety track could support this development and facilitate the submission of well characterized biosafety BioBricks to the iGEM Registry of Standard Biological Parts. Furthermore, since only the submission of parts (not the submission of engineered strains) to the Registry of Standard Parts is possible, iGEM teams are limited to the construction of plasmid-based biosafety devices. Enabling a controlled strain distribution alongside the BioBrick collection could facilitate the invention of even more sophisticated systems.

So, what comes next? Given that biotechnology is becoming more and more established in industry, complex biosafety systems that can efficiently prevent vertical as well as HGT are of high interest. Furthermore, GMOs designed to be deployed in open environments pose new biosafety challenges. An example for such applications can include the possible use of engineered microbes for bioremediation of chemically polluted areas [372, 373]. Given that the technological advancements over the last decades have led to a much deeper understanding of cellular biology and regulatory processes, it is likely that novel and more advanced biosafety approaches will be developed in the near future. Especially cell-free xenobiological systems would be of high interest as potential high-fidelity synthetic biosafety systems. Novel synthetic biosafety systems could even allow for safer deployment of GMOs in open environments.

In the future, we propose the construction of customized safety strains by harnessing the extensive knowledge collected through genome sequencing and functional annotation projects. On the one hand, pathway mapping could be used to find target genes which might circumvent certain auxotrophies. On the other hand, the identification of bottle necks in essential pathways for generating novel auxotrophies is feasible now. Moreover, basic research e.g. about mechanisms of HGT and bacterial evolution is needed to quantify the safety of previously described mechanisms more precisely.

## Abbreviations

aaRS: aminoacyl-tRNA synthetase
aTc: anhydrotetracycline
CeNA: Cyclohexene nucleic acid
CFPS: Cell-free protein synthesis
CMO: Chemically modified organism
CRISPR/Cas: Clustered regularly interspaced short palindromic repeats/CRISPR associated
CSP: Cold shock proteins
DAP: Diaminopimelic acid
GMO: Genetically modified organism
GNA: Glycerol nucleic acid
GRO: Genetically recoded organism
HGT: Horizontal gene transfer
HIV: Human immunodeficiency virus
HNA: Hexitol nucleic acid
iGEM: international Genetically Engineered Machine
MCP: Methyl-accepting chemotaxis protein
ncAA: non-canonical amino acid
NSAA: Non-standard amino acid
NTP: Nucleoside triphosphate
OTS: Orthogonal translation system
PAM: Protospacer-adjacent motive
QAPRTase: Chromosomal quinolinic acid phosphoribosyltransferase
RBS: Ribosome binding site
sAA: synthetic amino acid
sfGFP: super folder GFP
sgRNA: single-guide RNA
SLiDE: Synthetic auxotroph with ligand-dependent essential genes
TA: Toxin-antitoxin
TD: Threonine deaminase
TNA: Threose nucleic acid
xDNA: expanded DNA
XNA: Xeno DNA
yDNA: wide DNA
